# Peripheral blood lymphocyte subsets change after steroid withdrawal in renal allograft recipients: a prospective study

**DOI:** 10.1038/s41598-019-42913-4

**Published:** 2019-05-15

**Authors:** Laura Llinàs-Mallol, Dolores Redondo-Pachón, María José Pérez-Sáez, Dàlia Raïch-Regué, Marisa Mir, José Yélamos, Miguel López-Botet, Julio Pascual, Marta Crespo

**Affiliations:** 1 Department of Nephrology, Hospital del Mar, Barcelona, Spain; 2 Institute Hospital del Mar for Medical Research, Barcelona, Spain; 3 Department of Immunology, Hospital del Mar, Barcelona, Spain

**Keywords:** Medical research, Nephrology

## Abstract

Several studies have assessed clinical outcomes after steroid withdrawal (SW) in kidney transplant (KT) recipients, but little is known about its potential impact on lymphocyte subpopulations. We designed a prospective study to evaluate the long-term impact of SW in 19 KT recipients compared to 16 KT recipients without changes in immunosuppression (steroid maintenance, SM). We assessed renal function, presence of HLA antibodies and peripheral blood lymphocyte subsets at time of inclusion, and 3, 12 and 24 months later. The immunophenotype of 20 healthy subjects was also analyzed. Serum creatinine and proteinuria remained stable in SW and SM patients. SW did not associate with generation of *de novo* donor-specific antibodies. SW patients showed decreases in T-lymphocytes (p < 0.001), and in the CD4^+^ T cell subpopulation (p = 0.046). The proportion of B-lymphocytes (p = 0.017), and both naïve and transitional B cells increased compared to SM patients (p < 0.001). Changes in B cell subsets were detected 3 months after SW and persisted for 24 months. No changes were observed in NK cells related to steroid withdrawal. SW patients displayed significant changes in peripheral T and B cell subsets, transitioning to the phenotype detected in healthy subjects. This may be considered as a maintained positive effect of SW previously unnoticed.

## Introduction

Kidney transplantation is the best therapeutic option for patients with end-stage renal disease, given the improvement in quantity and quality of life compared with long-term dialysis^1–4^. Short-term graft-survival rate is over 95% one year after KT, but long-term survival is still limited, being the half-life around 10 years for kidney grafts from deceased donors^5,6^. Graft failure may occur due to loss of graft function or recipient death with a functioning graft^7^. Acute rejection in low-immunological risk KT recipients is below 15% with current immunosuppression, based on calcineurin inhibitors (CNI), antiproliferative agents such as mycophenolic acid (MPA) and steroids, and rarely produces graft-loss^8,9^. However, chronic rejection associated with donor-specific antibodies (DSA), also known as antibody-mediated rejection (AMR), has arisen as a major cause of graft loss, despite this immunosuppression strategy^7,10–12^. In the other hand, cardiovascular disease and cancer account for an excess of mortality in the transplant population^13–15^. Alternative drugs oriented to avoid CNI toxicity, such as mTOR inhibitors^9,16^ or CD80/CD86 blocker Belatacept™^17^, or minimization/withdrawal of some drugs have not achieved enough agreement to replace current immunosuppression.

Steroids are effective in reducing the incidence of acute rejection, but also a very frequent cause of undesirable effects and morbidity, with potential impact on graft survival^18,19^. Steroid withdrawal (SW) has been attempted for years by many groups, and the overall outcomes suggest that acute rejection rates increase, without clear advantages in morbidity and mortality^20–24^. Therefore, although SW is not generalized, it is applied to selected immunological low-risk KT recipients^25^ or in children, where advantage in growth is of utmost importance^26^. The lack of both long-term follow-up studies and evaluation of this treatment strategy on the development of chronic rejection are current limitations when assessing the interest of SW. In this setting, using immunological biomarkers as surrogates of immunological responses may be useful.

Although the role of DSA in promoting AMR and consequent graft loss has been well reported^27,28^, the influence of long-term SW in the development of anti-HLA DSA and AMR still remains unclear. Several studies have analyzed different peripheral blood lymphocyte (PBL) subpopulations in patients both before and after KT, finding some correlations with AMR. Higher percentages of T-effector memory lymphocytes correlate with increased risk of allograft dysfunction in stable KT recipients^29^, whereas patients with AMR show relevant presence of CD8^+^ CD28^−^T lymphocytes^30^. In a recent study, we identified a significant increase of NK cells expressing CD94/NKG2A receptor in patients with HLA DSA, especially in those with AMR^31^. Nevertheless, changes in immunosuppression may modulate the polarization of immune cell populations^32^. Few studies have evaluated the influence of SW on PBL, focused on SW short-term after KT. One study reported a decrease of NK cells under steroid maintenance compared to steroid avoidance^33^. Another report found no differences in peripheral blood lymphocytes between steroid withdrawal and steroid avoidance, in patients who received thymoglobulin as induction therapy^34^. A recent report that studies the association between potential tolerance biomarkers and immunosuppression, included the short-term results of a small cohort of patients who underwent steroid withdrawal early after KT for clinical reasons. The authors showed a significant increase in transitional B cells and no changes in T cells 3–6 months after steroid withdrawal^32^. However, there is no information regarding the influence of long-term SW on PBL subsets.

Based on these data, we designed an exploratory prospective study to analyze the long-term influence of SW on immunological biomarkers.

## Results

### Study population and clinical follow-up

**Figure 1:**
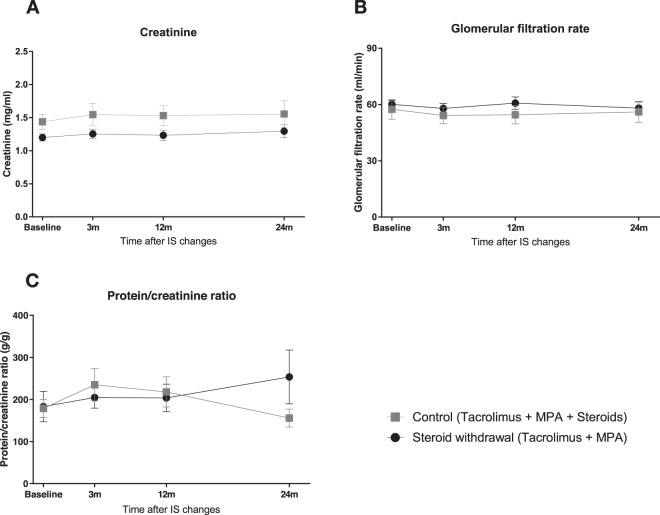
Both groups of the study maintained stable renal function along the follow-up, in terms of serum creatinine, eGFR and proteinuria. Plots show mean and standard error (SEM) for each study group.

Thirty-five patients with stable kidney function and without DSA were included in a prospective observational study: 19 withdrew steroids a median time of 19 months after transplantation (steroid withdrawal group, SW) and 16 maintained their baseline treatment, as a control group (steroid maintenance group, SM). Twenty healthy individuals were also immunophenotyped. All patients were followed up to 24 months. Main baseline characteristics of SW and SM patients are included in Table 1. Groups were similar in age, race, donor type, donor age, HLA mismatches, or previous acute rejection rates. The time post transplantation is detailed in Supplementary Fig. 1. A comparison between all lymphocyte subsets at baseline is shown in Supplementary Table 1. Patients in both groups showed stable renal function with no significant changes in serum creatinine, estimated glomerular filtration rate (eGFR) and proteinuria, measured as urinary protein/creatinine ratio during the study period (Fig. 1A–C). There was no graft loss or death along the study in any group. Furthermore, no patient in the steroid withdrawal group needed to reintroduce steroids.

**Table 1:** Baseline characteristics of the patients.

	Steroid withdrawal (N = 19)	Steroid maintenance (N = 16)	p - value
Recipient age (years) [mean (SD)]	53.0 (11.9)	52.4 (13.9)	0.89
Recipient gender (female) (n, %)	8 (42%)	3 (19%)	0.14
Race (caucasian) (n, %)	18 (95%)	14 (88%)	0.58
Type of donor (deceased) (n, %)	15 (79%)	15 (94%)	0.35
Donor age (years) [mean (SD)]	50 (12)	47 (16)	0.50
Time after KT (months) [median (p25–p75)]	19.15 (11.37–34.73)	17.03 (3.04–48.77)	0.73
Induction immunosuppression (antilymphocyte antibodies) (n, %)	3 (16%)	0	0.23
Delayed graft function (n, %)	4 (21%)	4 (25%)	1.00
Pre-inclusion acute rejection (n, %)	0	1 (6%)	0.46
**Sensitizing events before KT**			
Blood transfusions (n, %)	7 (37%)	2 (13%)	0.14
Previous pregnancies (n, %)	5/8 (63%)	1/3 (33%)	0.55
Retransplantation (n, %)	0	1 (6%)	0.46
**HLA mismatches**			
HLA mismatch Class I (A/B) [mean (SD)]	3 (1)	3 (1)	0.96
HLA mismatch Class II (DR) [mean (SD)]	1 (1)	1 (1)	0.59
**Panel reactive antibodies** ( **PRA** )			
Pre-KT PRA CDC > 5% (n, %)	0	0	NA
Peak PRA CDC > 5% (n, %)	4 (21%)	1 (6%)	0.61
**Antibodies**			
Anti-HLA DSA antibodies (n, %)	0	0	NA
Anti-HLA no DSA antibodies (n, %)	1 (5%)	0	1.00
**CMV status**			
D^+^/R^+^	11 (58%)	12 (75%)	0.48
D^+^/R^−^	5 (26%)	1 (6%)	0.19
D^−^/R^+^	2 (11%)	3 (19%)	0.64
D^−^/R^−^	1 (5%)	0	1.00
CMV infection (n, %)	4 (21%)	2 (13%)	0.67

Baseline characteristics of included patients. The table summarizes the baseline characteristics in patients who withdrew steroids (SW) and patients who maintained steroids (SM). *SD: Standard deviation*. *KT: Kidney transplantation*. *PRA: panel reactive antibodies*. *DSA: donor-specific antibodies*. *D: donor*. *R: recipient*.

### Steroid withdrawal did not promote the development of *de novo* DSA

No patient developed *de novo* DSA during the 24 months of sequential evaluation. Four patients developed *de novo* HLA no-DSA: two in the SW group (11%) and two in the SM group (13%) (p = 1.00). One patient in the SW group had HLA no-DSA class I and class II prior to SW and maintained these antibodies along the study.

### T cells decrease whereas B cells increase after steroid withdrawal

**Figure 2:**
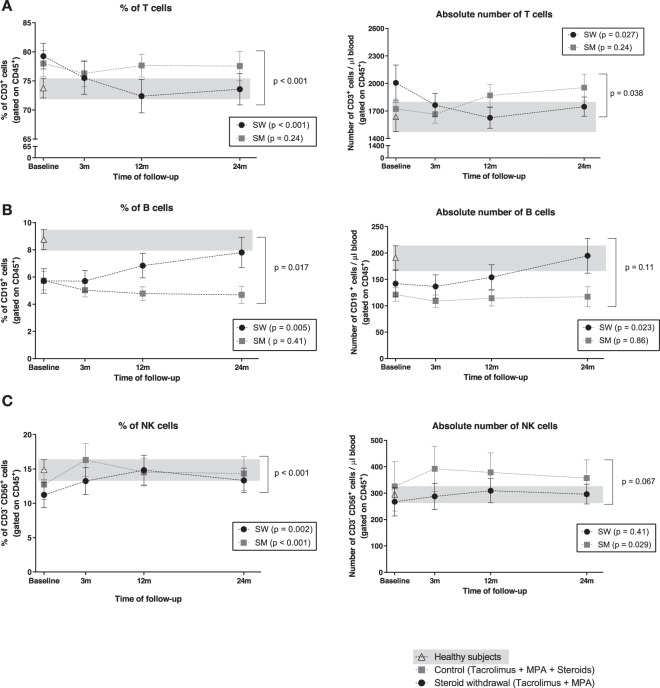
Evolution of T cells, B cells and NK cells percentages and absolute numbers after SW. Immunophenotyping analysis of (**A**) T cells (CD3^+^), (**B**) B cells (CD19^+^) and (**C**) NK cells (CD3^−^ CD56^+^) in patients before and after SW (black dots) and patients maintaining steroids (grey squares). HS data is depicted with white triangles and HS range is highlighted with a grey background. Dots show mean and SEM for each time point.

Patients who underwent SW showed a significant decrease in the percentage of circulating T cells during the first year of the study, followed by stabilization during the second year (baseline: 79.3 ± 9.6%, 12 months: 72.4 ± 12.6%, 24 months: 73.6 ± 11.4%; p < 0.001) (Fig. 2A). On the contrary, the SM group showed no changes in T cells along the study (p = 0.24). Evolution of T cells between the two groups was significantly different (p < 0.001). T cells from SW patients reached similar levels to those of healthy subjects, in contrast to the SM group (Fig. 2A). This effect was also observed when measuring absolute numbers (Fig. 2A, SW p = 0.027; SM p = 0.24; between groups p = 0.038).

The proportion of B cells increased during the follow-up in SW patients (baseline: 5.7 ± 3.9%, 24 months: 7.8 ± 4.8%, p = 0.005) (Fig. 2B), but not in SM group (p = 0.41). Evolution of B cells between groups was significantly different (p = 0.017). Twenty-four months after SW, the proportion of B cells reached the level of healthy subjects, but the SM group did not (Fig. 2B). Absolute numbers of B cells behaved similarly (SW p = 0.023; SM p = 0.86) (Fig. 2B).

The NK cell percentage increased significantly within the first year and stabilized afterwards in both groups (SW p = 0.002; SM p < 0.001, Fig. 2C). The evolution was different between groups, with the highest peak reached by the SM group at three months (p < 0.001, Fig. 2C). No differences due to SW could be identified in NK cell subsets considering the expression of NKG2A^+^, NKG2C^+^ (Supplementary Fig. 2A,B), ILT2^+^, KIR^+^ and CD161^+^ (data not shown).

### Steroid withdrawal promotes a decrease of CD4^+^ T cells

**Figure 3:**
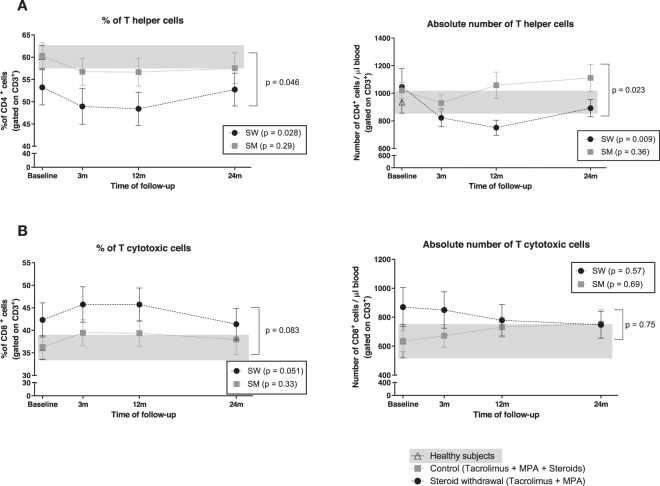
Lower percentage and absolute numbers of CD4^+^ T cells after SW. Immunophenotyping analysis of percentage and absolute numbers of (**A**) CD4^+^ T cells (CD3^+^CD4^+^) and (**B**) CD8^+^ T cells (CD3^+^CD8^+^) in patients before and after SW (black dots) and maintaining steroids (grey squares). HS data is depicted with white triangles and HS range is highlighted with a grey background. Dots show mean and SEM for each time point.

In order to understand differences observed on T cells, we analyzed T cell subsets. CD4^+^ T cells decreased significantly during the first year after SW (baseline: 53.2 ± 17.2%, 12 months: 48.4 ± 16.3%) and returned to baseline thereafter (24 months 52.7 ± 15.6%) (Fig. 3A). The two groups of treatment had a different evolution of both proportion (p = 0.046) and absolute numbers (p = 0.023) of CD4^+^ T cells (Fig. 3A). CD8^+^ T cells did not display significant changes (Fig. 3B).

### Higher percentages of B cells after steroid withdrawal are mainly due to an increase of naïve B cells

**Figure 4:**
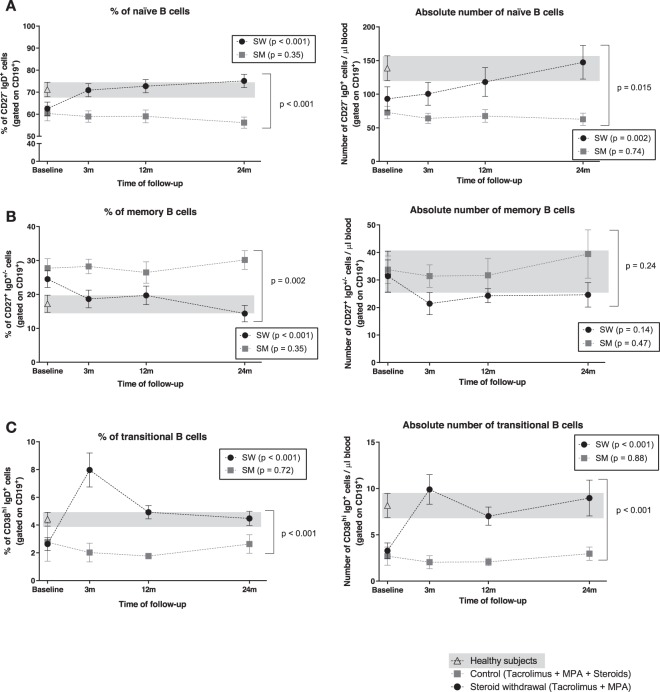
Evolution of B cell subsets percentages and absolute numbers after SW. Immunophenotyping analysis of percentage and absolute numbers of (**A**) naïve B cells (CD19^+^ CD27^− ^IgD^+^), (**B**) memory B cells (CD19^+^ CD27^+^ IgD^+/−^) and (**C**) transitional B cells (CD19^+^ CD38^hi^ IgD^+^) in patients before and after SW (black dots) and patients maintaining steroids (grey squares). HS data is depicted with white triangles and grey background corresponds to HS range. Plots show mean and SEM for each time point.

Analysis of B cell subsets identified a significant increase of naïve B cells after SW (baseline: 62.5 ± 13.2%, 24 months: 75.1 ± 12.9%; p < 0.001), in contrast to SM group (p = 0.35), being the evolution significantly different between groups (p < 0.001) (Fig. 4A). Absolute numbers of naïve B cells behaved similarly (SW p = 0.002; SM p = 0.74; between groups p = 0.015) (Fig. 4A). In SW patients, but not in SM patients, both percentages and absolute numbers of naïve B cells reached similar levels to those of healthy subjects (Fig. 4A).

Regarding memory B cells, SW patients showed significantly lower percentages along the follow-up (p < 0.001) compared to SM patients (p = 0.35) (between groups p = 0.002) (Fig. 4B). Absolute numbers did not reflect these changes (SW p = 0.14; SM p = 0.47; between groups p = 0.24) (Fig. 4B). The analysis of naïve and memory B cell subsets using a different immunophenotyping strategy based on CD38 and IgD expression^35^ showed results consistent with the CD27 and IgD staining (data not shown).

### Steroid withdrawal promotes an increase of circulating transitional B cells

**Figure 5:**
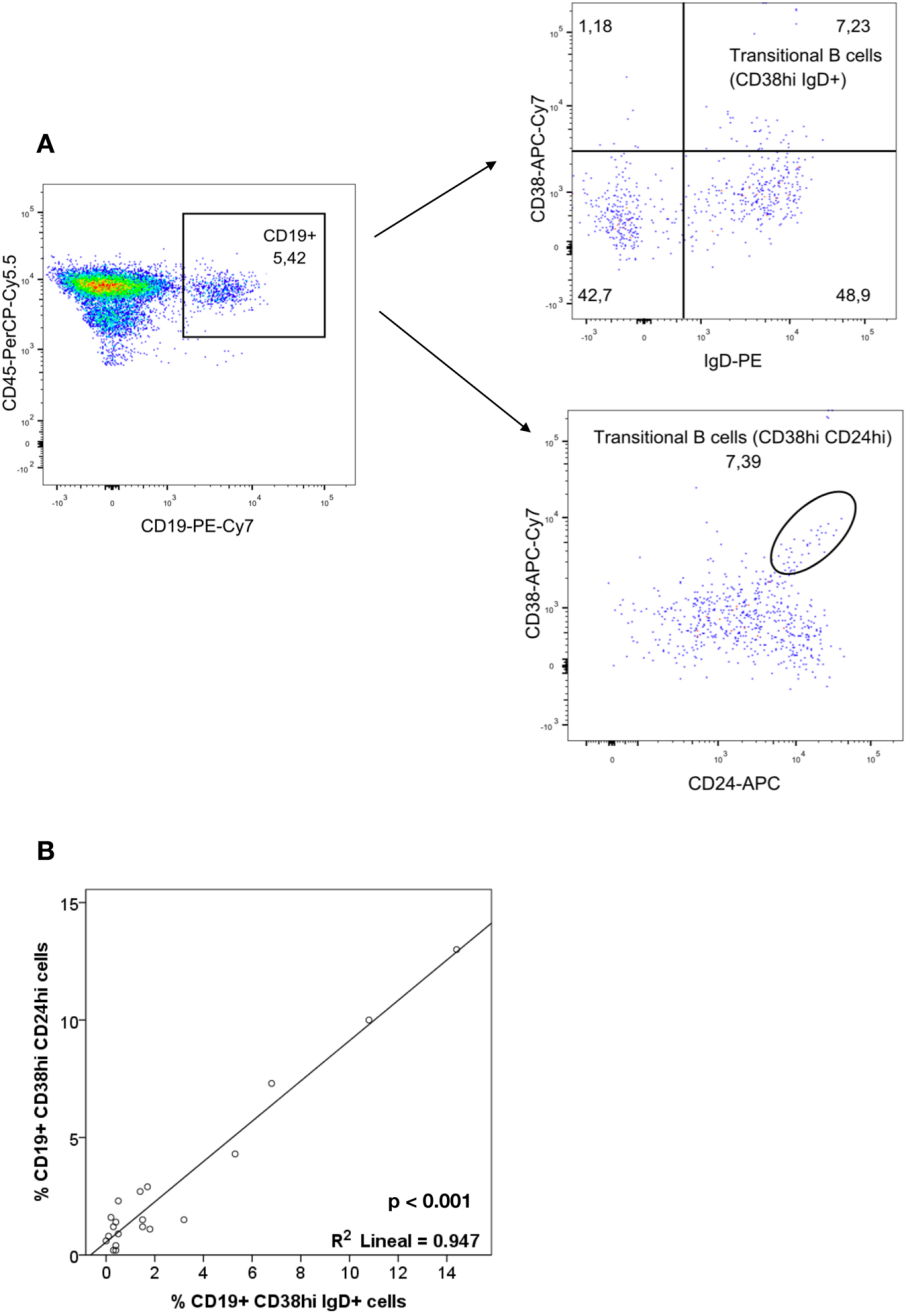
Characterization of transitional B cells. (**A**) Representative flow cytometry plots from the same patient, including or not CD24 staining. (**B**) Correlation graph and Pearson correlation value for the CD19^+^CD38^hi^IgD^+^ and CD19^+^CD24^hi^CD38^hi^ populations.

We found a significant increase in the percentage of transitional B cells three months after SW (baseline: 2.6 ± 1.9%, 24 months: 4.5 ± 2.2; p < 0.001) compared to SM group (p = 0.72) (Fig. 4C). Absolute numbers showed similar results (Fig. 4C). SW and SM patients displayed a significantly different evolution in both the proportion and absolute numbers of transitional B cells (both p < 0.001). Moreover, SW patients presented similar numbers to those of healthy subjects (Fig. 4C). In order to conform the bibliographic evidence^36–39^ supporting current transitional B cell characterization as CD19^+^CD24^hi^CD38^hi^ cells, we assessed the percentage and absolute numbers of transitional B cells from 20 KT recipients including a CD24 immunophenotyping strategy (Fig. 5A). Our results indicate a strong correlation between CD19^+^CD38^hi^IgD^+^ and CD19^+^CD24^hi^CD38^hi^ populations (Pearson correlation value 0.947, p < 0.001) (Fig. 5B).

## Discussion

Although steroid avoidance has been associated with increased risk of acute rejection^20–24^, there is growing evidence that SW may offer several advantages in low risk KT patients^22^. Our results support that SW can be accomplished in immunologically low-risk KT patients without compromising renal function the first two years after steroid withdrawal. Interestingly, Haller *et al*. have recently suggested that SW may be safe if it is undertaken beyond 18 months after KT^24^. The median post-KT time of our cohort at SW was 19 months. We found that patients who underwent steroid withdrawal maintained stable renal function without appearance of *de novo* anti*-*HLA DSA and they showed decreased circulating T CD4^+^ cells and increased naïve and transitional B cells, a similar immunophenotype profile to the one of healthy subjects.

Steroid withdrawal in low-risk KT recipients has been shown to be safe in terms of *de novo* DSA development^40–42^. Two studies showed no influence of early or mid-term SW on *de novo* DSA development^40,41^. In agreement, our long-term SW patients did not show *de novo* HLA DSA antibody during two years of follow-up.

The effect of SW on peripheral T cells is less clear in the literature. A recent study^32^ reported no significant differences when comparing T cell percentages before and 3–6 months after SW. However, the fact of doing a longer follow-up allowed us to see a significant decrease in total T and CD4^+^ T subset in the SW group. This decrease seems to be restricted to the first year after SW, although CD4^+^ T cell numbers did not recover baseline levels. In contrast, the percentage and absolute numbers of CD8^+^ T cells did not significantly change in the SW group, although dynamics of CD8^+^ T cells percentages reflected the changes of CD4^+^ T cells percentages. This supports the hypothesis that SW promotes a reduction of circulating CD4^+^ T cells in KT patients. The role of glucocorticoids on the thymopoiesis remains uncertain, but there is evidence supporting that steroids promote the differentiation of Th2 over Th1 cells^43^. Further studies considering specific CD4^+^ T cell subpopulations may clarify this particular issue.

To our knowledge, few studies have explored B cell dynamics before and after SW^32–34^. These studies did not describe results of naïve and memory B cell subsets. Our cohort of KT recipients after long-term SW experienced a significant increase in total B cells without an increase in memory B cells, potentially related to antibody production. The B cell increase was attributable to a greater number of circulating naïve B cells, which do not secrete immunoglobulins^44^ and are more sensitive to glucocorticoid-induced apoptosis^43,45^. This may explain the observed relative reduction in the proportion of memory B cells but not their absolute numbers. Our results demonstrate for the first time that SW promotes a gradual increase in naïve B cells in KT recipients along the follow up, reaching the levels of healthy subjects. Regarding transitional B cells, patients under conventional immunosuppression (based on CNI, mycophenolic acid and steroids) have shown significantly lower levels than healthy subjects^46^. On the other hand, Rebollo-Mesa *et al*. found that the percentage of transitional B cells increased in KT recipients 3 to 6 months after SW^32^. Remarkably, we have also found a striking increase of transitional B cells 3 months after SW, reaching similar levels to heathy subjects after one year, whereas SM patients maintained stable numbers during the follow-up. This effect may also be due to the sensitivity of early mature B cells to glucocorticoid-induced apoptosis^43,45^.

The expansion of B cells and differential expression of B cell-related genes have been described as biomarkers of tolerance in previous studies^47–49^. Our results show that the B cell immunophenotype 1-year after SW resembles the one expressed by healthy subjects. These results suggest that steroid treatment in KT recipients is disrupting normal B cell subset distribution^45^. Immunosuppression has been related not only to a distinct lymphocyte distribution but also to their gene expression in peripheral blood^32^. The estimated genetic probability of tolerance increased after SW, independently of the peripheral expansion of transitional B cells. The important function that transitional B cells may play in transplantation tolerance arises as an open question, connected to their potential anti-inflammatory function. “Operational tolerance” has been hypothesized to be an immunological state of not recognizing the graft as “foreign”, independently of the immunosuppression treatment, rather than a condition in which inflammatory processes are controlled by steroids or other immunosuppressive treatments^50^.

The small sample size and the absence of graft-biopsies do not permit to evaluate whether the observed changes in PBL correlate with either tolerance or subclinical damage, and therefore the influence on graft outcomes. However, we present the results of the first exploratory study with PBL analysis long-term after SW in a homogeneous clinically stable KT group of patients. Functional studies could be of interest to understand the pathophysiological consequences of SW on the peripheral distribution of T and B cell subsets.

In summary, our study confirms that SW in low-risk KT recipients is safe in terms of renal function and does not associate with the generation of *de novo* DSA. SW leads to a distinct peripheral lymphocyte distribution in KT patients, which resembles that of healthy subjects. These changes are similar to those described in tolerant KT patients and opposite to the profile found in KT patients with chronic rejection.

## Materials and Methods

### Study design and population

This is a prospective observational study where we evaluated the potential influence of immunosuppression changes in the development of HLA antibodies and the distribution of PBL subsets in 35 patients who underwent kidney transplantation at Hospital del Mar, Spain. KT recipients with stable renal function, without proteinuria and DSA who withdrew steroids (SW) per clinical practice at the out-patient clinic were included from June 2011 to September 2015. On the other hand, a parallel group of patients who maintained steroids (SM) were included as a control group.

Initially, all patients received 5 mg of prednisone/day, together with tacrolimus (mean trough blood level 7.7 ng/mL) and mycophenolate acid (mean daily dose 570 mg). At last follow-up tacrolimus trough blood levels were similar in both groups (SW 6.8 ng/mL and SM 6.49 ng/mL) and mycophenolate acid dose stayed stable (SW 508 mg/day and SM 566 mg/day). Clinical evaluation (serum creatinine, eGFR and proteinuria), HLA antibody analysis and PBL immunophenotyping were performed before and 3, 12 and 24 months after SW or inclusion. In addition, PBL subsets of 20 healthy subjects were analyzed. The study was approved by the CEIC Parc de Salut Mar Ethical Research Board (2011/4385/I) and all patients signed informed consents. The clinical and research activities being reported are consistent with the Principles of the Declaration of Istanbul and the Declaration of Helsinki. No organs were procured from prisoners.

### Determination of HLA antibodies

Serum samples were collected and stored at −80 °C until analysis. Screening for anti-HLA antibodies was performed with Luminex Lifecodes LifeScreen Deluxe assay (*Gen-probe*®, Stanford), and anti-HLA alloantibody identification was performed using Lifecodes LSA Class-I (93 beads) and/or Class-II (84 beads) assays (*Gen-probe*®, Stanford), as previously described^51^. Donor HLA antibody specificity was ascribed following the results of single antigen assay, considering donor HLA typing or linkage disequilibrium for C or DQ antigens when typing was not available. A reaction with mean immunofluorescence intensity over 1000 was considered positive.

### Immunophenotyping analysis

**Figure 6:**
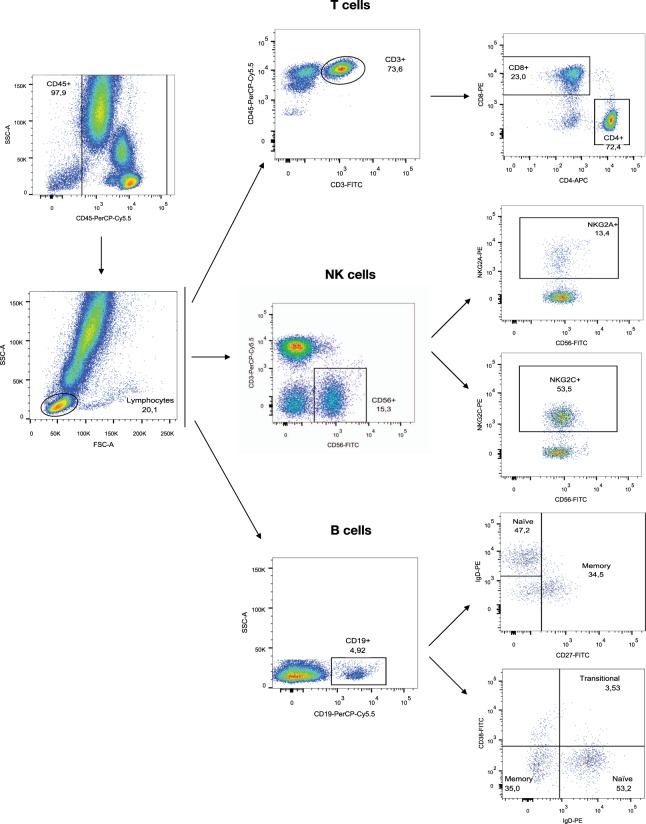
Gating strategy for studying PBL subpopulations. Representative flow cytometry plots from the same patient, illustrating the gating strategy used for the study. (**Above**) Gating strategy for T cell tube. (**Middle**) Gating strategy for NK cell tube. (**Below**) Gating strategy for B cell tube.

Immunophenotyping was performed by flow cytometry on fresh peripheral blood samples, obtained by venous puncture in EDTA tubes. Samples were pretreated with saturating concentrations of human aggregated immunoglobulins to block FcγR and then labeled with different antibody combinations to define T and B lymphocytes, and NK-cell subsets in separated tubs (Supplementary Table 2 and Fig. 6). Samples were acquired by a FACS Canto II cytometer and data analyzed by FACS Diva and FlowJo softwares (BD Biosciences™). T lymphocytes were characterized as CD3^+^ cells, T helper as CD3^+^CD4^+^cells and T-cytotoxic as CD3^+^CD8^+^ cells. B lymphocytes were characterized as CD19^+^ cells and subpopulations were analyzed considering IgD and either CD27 or CD38 expression^35^: naïve (CD27^−^IgD^+^ or CD38^−^IgD^+^), memory (CD27^+^IgD^+/−^ or CD38^−^IgD^−^) and transitional B cells (CD38^high^IgD^+^)^35^. NK cells were identified as CD3^−^CD56^+^ cells and subsets were defined by expression of NKG2A, NKG2C, ILT2, KIR or CD161. Absolute numbers of cells were calculated from parallel blood counts and percentages of subsets referred to total CD3^+^, CD19^+^ or CD3^−^CD56^+^ cells respectively, which in turn are referred to total lymphocytes. Validation of transitional B cell immunophenotype was performed comparing the CD19^+^CD38^hi^IgD^+^ and CD19^+^CD24^hi^CD38^hi^ gating strategies in 20 KT recipients from another cohort (Fig. 5A,B).

### Statistical analysis

Comparisons between normally distributed variables were carried out by using Student’s t-test and non-parametric variables were analyzed with U Mann-Whitney test. Normal distribution of continuous variables was tested with Kolgorov-Smirnoff and Shapiro-Wilk tests. Chi-squared or Fisher’s exact tests were used for dichotomous variables. Generalized Estimating Equations (GEE) population-averaged model was used for analyzing changes in PBL subpopulations, including an interaction term in order to check differences between study groups. Two p-values were obtained, one for each study group and PBL subpopulation evolution (therefore representing the comparison between baseline and 3, 12 and 24 months data) and another one evaluating the differences in the evolution of each PBL subpopulation between the two groups of study. A p value < 0.05 was considered statistically significant. We have further performed an alternative analysis with a repeated measures ANOVA test for PBL subpopulations (Supplementary Table 3). Statistical analysis was performed using SPSS^®^ v.22.0 (IBM Corp, New York, USA) and Stata^®^ v.15 (STATA Corp, Texas, USA) for clinical and cellular data respectively.

## Supplementary information

Supplementary Information

## Data Availability

The datasets generated during and/or analyzed during the current study are not publicly available because they have not been uploaded in a public database but are available from the corresponding author on reasonable request.
