# Supplementary material for: Peripheral blood lymphocyte subsets change after steroid withdrawal in renal allograft recipients: a prospective study
Source: Sci Rep. 2019 May 15;9:7453. doi: 10.1038/s41598-019-42913-4 (PMC6520389; doi:10.1038/s41598-019-42913-4)
Supplement: Supplementary file 1 — Supplementary Information [file 41598_2019_42913_MOESM1_ESM.pdf]

Supplementary Information

Peripheral blood lymphocyte subsets change after steroid withdrawal in renal allograft recipients: a prospective study

Laura Llinàs-Mallol[1,2] MSc, Dolores Redondo-Pachón[1,2] MD PhD, María José Pérez-Sáez[1,2] MD PhD, Dàlia Raïch-Regué[1,2] PhD, Marisa Mir[1] MD, José Yélamos[2,3] PhD, Miguel López-Botet[2,3] PhD, Julio Pascual[1,2 # *] MD PhD and Marta Crespo[1,2 #] MD PhD.

[1]Department of Nephrology, Hospital del Mar, Barcelona, Spain.

[2]Institute Hospital del Mar for Medical Research, Barcelona, Spain.

[3]Department of Immunology, Hospital del Mar, Barcelona, Spain.

* Corresponding author. E-mail: jpascual@psmar.cat

Senior authors

This PDF file includes Figures S1-S2 and Tables S1-S3

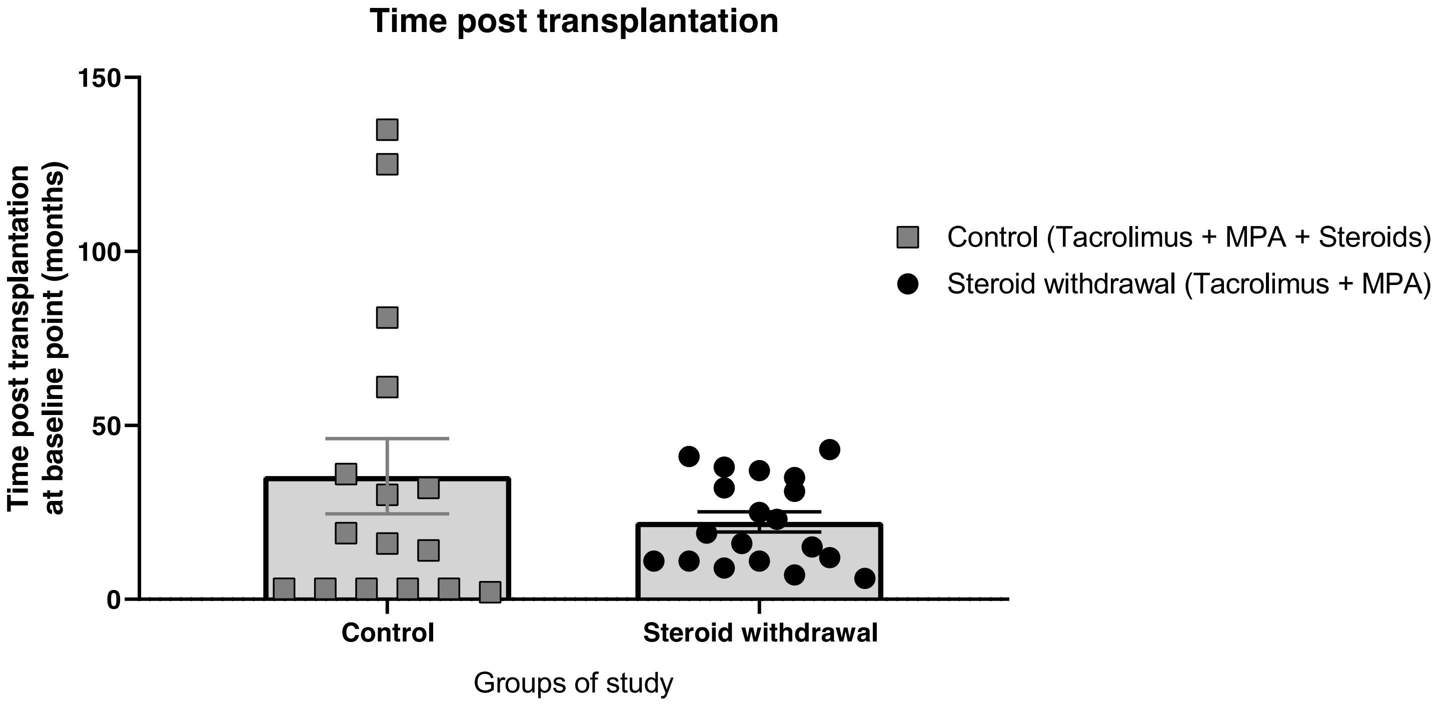

Supplementary Figure 1. Time post transplantation for each of the 35 patients at the baseline point of the study. Black dots represent patients who withdrew steroids and grey squares patients that maintained steroids.

A

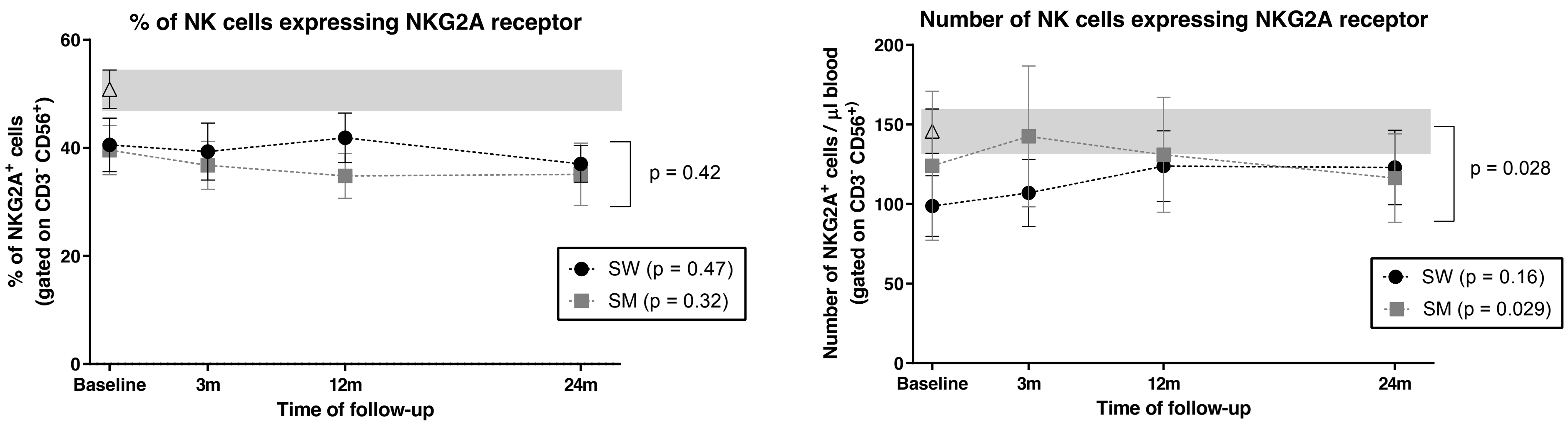

B

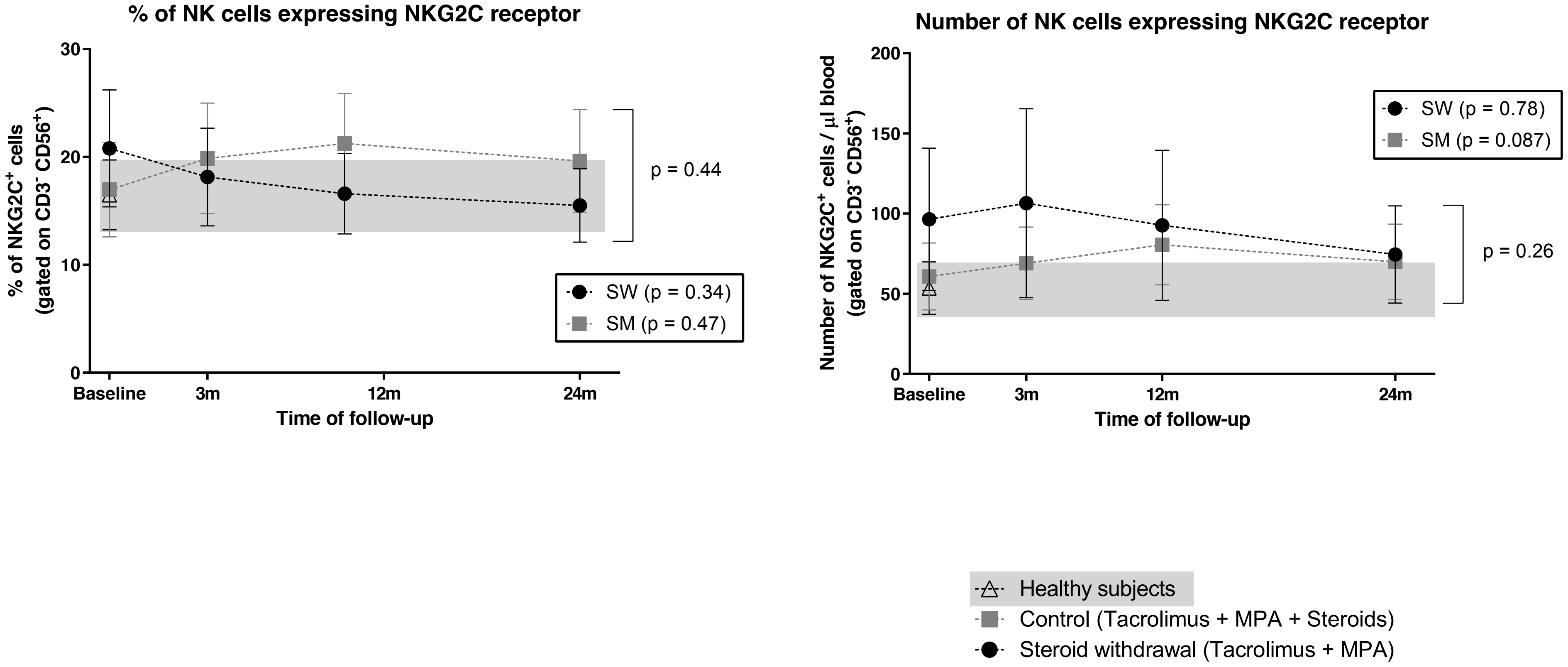

Supplementary Figure 2. Evolution of NK cell subsets NKG2A$^+$ and NKG2C$^+$ percentages and absolute numbers after SW. Immunophenotyping analysis of percentage and absolute numbers of **(A)** NKG2A$^+$ NK cells (CD3$^-$ CD56$^+$ NKG2A$^+$) and **(B)** NKG2C$^+$ cells (CD3$^-$ CD56$^+$ NKG2C$^+$) in patients before and after SW (black dots) and patients maintaining steroids (grey squares). HS data is depicted with white triangles and grey background corresponds to HS range. Plots show mean and SEM for each time point.

Supplementary Table 1. Values of lymphocyte subsets at baseline time. Values of each lymphocyte subset are expressed in mean and standard error of mean (SEM) and p-values are obtained from a Mann-Whitney-U-test.

	**Steroid withdrawal (N = 19)**	**Steroid maintenance (N = 16)**	**p - value**
Total lymphocytes	2564 (267)	2246 (181)	0.40
% T cells	79.3 (2.2)	78.0 (2.3)	0.71
Absolute number of T cells	2007 (194)	1723 (107)	0.27
% T helper cells	53.2 (4.0)	60.3 (3.0)	0.19
Absolute number of T helper cells	1046 (133)	1020 (59)	0.59
% T cytotoxic cells	42.3 (3.8)	36.3 (2.7)	0.29
Absolute number of T cytotoxic cells	870 (135)	636 (73)	0.27
% B cells	5.7 (0.9)	5.7 (0.7)	0.59
Absolute number of B cells	142 (25)	121 (13)	0.94
% of naïve B cells	62.5 (3.0)	60.3 (3.4)	0.71
Absolute number of naïve B cells	93 (18)	73 (9)	0.91
% of memory B cells	24.5 (2.5)	27.7 (2.8)	0.22
Absolute number of memory B cells	31 (6)	34 (5)	0.40
% of transitional B cells	2.6 (0.5)	2.8 (1.4)	0.10
Absolute number of transitional B cells	3 (1)	3 (1)	0.24
% NK cells	12.4 (2.1)	12.8 (2.2)	0.94
Absolute number of NK cells	369 (114)	326 (94)	1.00
% of NKG2A$^+$ NK cells	39.4 (4.8)	39.6 (4.6)	0.94
Absolute number of NKG2A$^+$ NK cells	115 (24)	124 (47)	0.94
% of NKG2C$^+$ NK cells	21.7 (5.2)	17.0 (4.4)	0.78
Absolute number of NKG2C$^+$ NK cells	96 (44)	61 (21)	0.78

Supplementary Table 2. Antibodies used in the study. The table summarizes antigen, clone, fluorochrome and company for each antibody used in the study.

Antigen	Clone	Fluorochrome	Company
CD3	SK7	FITC / APC / PerCP-Cy5.5	BD Biosciences™
CD4	SK3	APC / PerCP-Cy5.5	BD Biosciences™
CD8	SK1	PE	BD Biosciences™
CD19	SJ25C1	PerCP-Cy5.5	BD Biosciences™
CD27	L128	FITC	BD Biosciences™
CD38	HB-7	FITC	BD Biosciences™
CD45	2D1	PE / APC / PerCP-Cy5.5	BD Biosciences™
CD56	NCAM 16.2	FITC	BD Biosciences™
CD161	HP-3G10	PE	Generated in our lab
IgD	IA6-2	PE	BD Biosciences™
NKG2A	Z199	PE	Provided by Dr. A. Moretta
NKG2C	MAB1381	PE	R&D systems™
ILT2	HP-F1	PE	Generated in our lab
KIR	5.133, CH-L, DX9, HP-3E4	PE	Provided by Drs. M. Colonna; S. Ferrini and L. L. Lanier. HP-3E4 was generated in our lab
F(ab')2 goat anti-mouse IgG + IgM		PE	Jackson ImmunoResearch™

Supplementary Table 3. Results from repeated measures ANOVA test. Table depicts the results from an alternative analysis with a repeated measures ANOVA test for PBL subpopulations.

	Steroid maintenance	Steroid withdrawal	Joint p-value
Total lymphocytes	0.20	0.33	0.23
% T cells	0.60	< 0.001	< 0.001
Absolute number of T cells	0.10	0.006	0.006
% T helper cells	0.23	0.06	0.08
Absolute number of T helper cells	0.32	0.003	0.008
% T cytotoxic cells	0.38	0.15	0.21
Absolute number of T cytotoxic cells	0.41	0.33	0.39
% B cells	0.08	< 0.001	< 0.001
Absolute number of B cells	0.84	< 0.001	0.002
% of naïve B cells	0.28	< 0.001	< 0.001
Absolute number of naïve B cells	0.68	< 0.001	< 0.001
% of memory B cells	0.58	< 0.001	0.002
Absolute number of memory B cells	0.54	0.22	0.36
% of transitional B cells	0.76	< 0.001	< 0.001
Absolute number of transitional B cells	0.88	< 0.001	< 0.001
% NK cells	0.01	0.005	< 0.001
Absolute number of NK cells	0.21	0.62	0.39
% of NKG2A$^+$ NK cells	0.08	0.50	0.16
Absolute number of NKG2A$^+$ NK cells	0.40	0.34	0.39
% of NKG2C$^+$ NK cells	0.26	0.10	0.12
Absolute number of NKG2C$^+$ NK cells	0.36	0.74	0.61
